# Phleboliths of venous malformation studied with scan electron microscopy. Case report and literature review

**DOI:** 10.4317/jced.61738

**Published:** 2024-07-01

**Authors:** Norma-Guadalupe Ibáñez-Mancera, Eric Partida-Rodríguez, Oswaldo Partida-Rodríguez

**Affiliations:** 1 Centro Interdisciplinario de Ciencias de la Salud UST, Instituto Politécnico Nacional, CDMX, México; 2 Servicio de Cirugía Maxilofacial del Centro Médico “Lic. Adolfo López Mateos” ISEM, México. Facultad de Odontología Universidad Autónoma del Estado de México, México; 3 Unidad de Medicina Experimental, Facultad de Medicina, Universidad Nacional Autónoma de México, México. Hospital General de México, CDMX México

## Abstract

Vascular anomalies are classified in two categories, vascular tumors and vascular malformations. Among the latter, venous malformations are the second most common vascular anomalies. In addition to pain and/or increase of volume, venous malformations can lead to phlebolith formation with time. We present the case of a female patient of 17 years of age, with a tumoration of four centimeters of diameter, located in the submentonian region, with nine calcified foci identified by three-dimension reconstruction. The calcifications were studied with scanning electron microscopy, detecting mainly the presence of carbon, oxygen and calcium. Regarding the tissue organization, it was possible to observe the development of filamentous aggregates of carbon macroparticles. As an important part of the evaluation for diagnostics and for the treatment of vascular anomalies, it is necessary to do a complete clinical history, and the clinic evaluation of the lesion should be complemented with imagenology studies. This will allow identification of the size and extension of the lesion and the presence of calcifications, which must be considered as a presumptuous diagnosis to vascular lesion.

** Key words:**Vascular anomalies, vascular tumors, vascular malformations, phlebolith.

## Introduction

Vascular anomalies are more common during childhood, occurring in one of every 22 children and 40% of venous malformations appear in head and neck (1,2). They are more common in men than women, with a ratio of 2.5:1 (3). They are present more frequently from birth and their progression is generally proportional to the child growth, however, sometimes can present an excessive growth (4).

The International Society for the study of Vascular Anomalies classifies vascular lesions based on the histological characteristics and the clinical course and the treatment into two categories: vascular tumors and vascular malformations (5,6). Vascular tumors are subclassified: benign, locally aggressive, or borderline and malignant. Vascular malformations are subclassified: Simple (low-flux and high-flux, combined, of major named vessels and associated with other anomalies. This classification allows a clear diagnosis and determination of therapeutic possibilities for these alterations (5).

To diagnose vascular anomalies, especially in pediatrics, it is necessary to evaluate the following characteristics: age of start, growth pattern in relation to the patient development, presence of pain or pruritus, hemorrhage, multiple lesions, family and personal background, size, fluctuation and location (2).

Vascular tumors have been reported as the most frequent vascular anomalies in pediatric population. One study described a vascular tumor frequency of 75.4%, whereas the vascular malformations were observed in 24.6% of cases (3). Similar results were reported in another study, where 60.5% of vascular anomalies corresponded to vascular tumors and the remaining 39.5 % to vascular malformations (4). The most common vascular anomaly, hemangioma, is a congenital vascular benign tumor which is presented in children. These tumors consist of the proliferation of blood vessels that generally involute with time. Regularly, they are easily observed clinically, but hard to identify when they are intraosseous or intramuscular (5,7).

Regarding vascular malformations, they can be either deep or superficial. The most superficial are violaceous. The diagnostics of deep ones is complicated, because they are not visible until they reach a size that would increase the volume in the zone. Consequently, it is necessary to get support with image studies. Within the group of vascular malformations, the venous malformations are the most common (8,9).

The vascular malformations are classified in four main groups: capillary malformations, venous malformations, lymphatic malformations, and arteriovenous malformations (10). Venous malformations have an incidence of 1 in 10,000 (9,11,12) and they constitute 40% to 65% of vascular anomalies. There is no gender predilection and regularly they are present since childhood in 0.3% of children (2,6,13). They are more common in head and neck being visible since birth, however, in some cases they are not identified due to their location in deep tissue (11).

The venous malformations are low-flux vascular anomalies composed by channels of venous ectasia (2,11). They can include capillary, veins and lymphatic malformations (6), and can develop into soft tissue, with the possibility to produce asymmetry and deformation and even incapacity (2). They are generally unifocal lesions (12) and presented spontaneously, but in some cases, they can be multiple or familial (11). The hemangiomas grow with the child and involute, on the contrary, venous malformations commonly increase their size during puberty (2).

Clinically, venous malformations are depressible or fluctuating, but not pulsatile lesions. When they are superficial lesions, they look blackish, violaceous, or bluish, but they can also involve deep tissue, such as bone, muscle and subcutaneous plains (14), hence the color will be paler. In some cases, they can look elevated, and pain is the second most common symptom next to the presence of the tumoral mass. They can be well located or be extended lesions (2,11,13) and, with time, they can produce pain and/or volume increase. Little clots can be formed secondary to trauma or venous stasis, which can promote the formation of phleboliths (2,6,11,12). The presence of phleboliths can facilitate radiographic diagnosis (9).

Phleboliths are thrombotic calcifications that can be easily observed with the imagenology studies used for the evaluation of the lesion (11). Radiographically, they are observed as round calcified masses (6). The presence of phleboliths is very suggestive of hemangiomas and vascular malformations. There are studies that report their presence, ranging from 2 and 3% to 15% and 25% (14) of the venous malformations cases. If it is necessary to distinguish them from a sialolyte, it is recommended to take a sialograph.

The treatment for venous malformations depends on the location and the symptomatology. There are several other considerations including: the kind of venous malformation, its location, size, extension and deepness, as well as factors regarding the patient condition, such as age. In the case of small lesions, treatment is not necessary, just keep the patient under observation, whereas big lesions do require a specific treatment (2,12,12). Vascular dynamics must be also considered (15).

There are several options for the treatment of vascular malformations, including conservative management, pharmacologic treatment, intervention of minimal invasion, radiotherapy, laser therapy and conventional surgery (2,). Among the options for the treatment of venous malformations, there are laser therapy, sclerotherapy, embolization, electrocauteration, steroid administration and surgical removal (1,12), surgery being the most common elected therapy followed by sclerotherapy (9,14,15).

## Case Report

**Figure 1:**
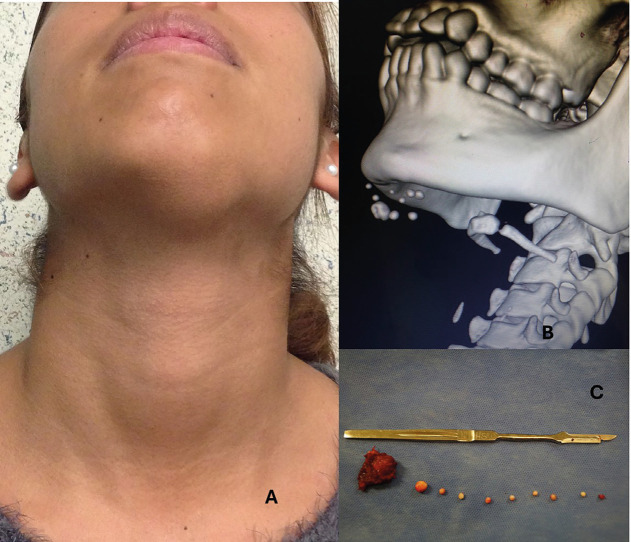
A) Clinical aspect of the tumor. B) Tridimensional reconstruction. C) Macroscopic aspect of the mineralization, spherical calcifications (phleboliths).

A 17-year-old female patient is presented, with a tumoration of 4 cm in diameter, located in the submentonian region (Fig. 1A). The patient referred two years of evolution with a slow growth and pain at palpation. She received a previous treatment with antibiotics and anti-inflammatory drugs under the supposition of an infectious process.

An exploratory puncture was made, obtaining bloody material and observing an immediate reduction in the size of the tumor. In the computed axial tomography (CAT) scan, it was identified a hypodense lesion with multiple circular hyperdense foci, the largest one with a size of 6 mm and the smallest one with 2 mm in diameter (Fig. 1B). A 3D reconstruction was made noticing 9 calcified foci. When the data from the clinical evaluation, the presence of blood in the aspiration and the presence of calcifications was integrated, the diagnosis reached was: Vascular anomaly associated to phleboliths.

The treatment started with the infiltrative application of a sclerosing agent (3% polidocanol) once a week for three months. After this time the lesion went from fluctuating to firm and the size diminished radically (approximately 60%). The enucleation was made under general anesthesia, obtaining a very fibrotic mass with nine spherical calcifications (phleboliths) of varying sizes (Fig. 1C). The histopathological result was compatible with venous malformation.

**Figure 2:**
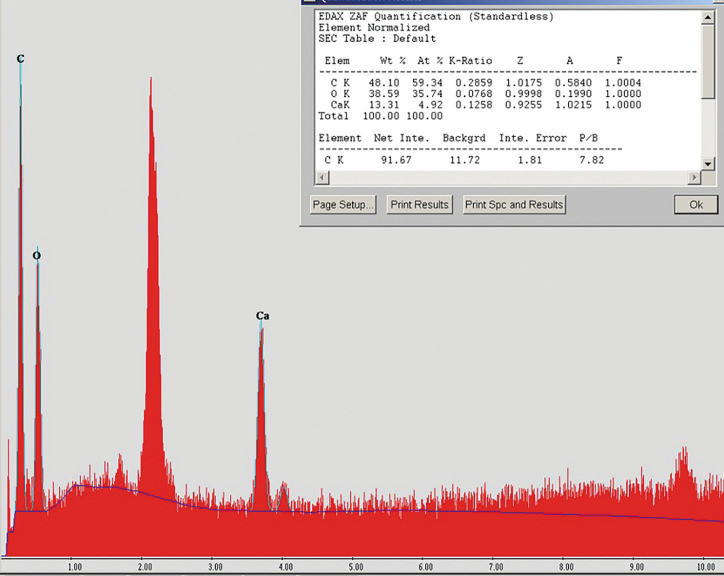
Chemical composition of phleboliths.

**Figure 3:**
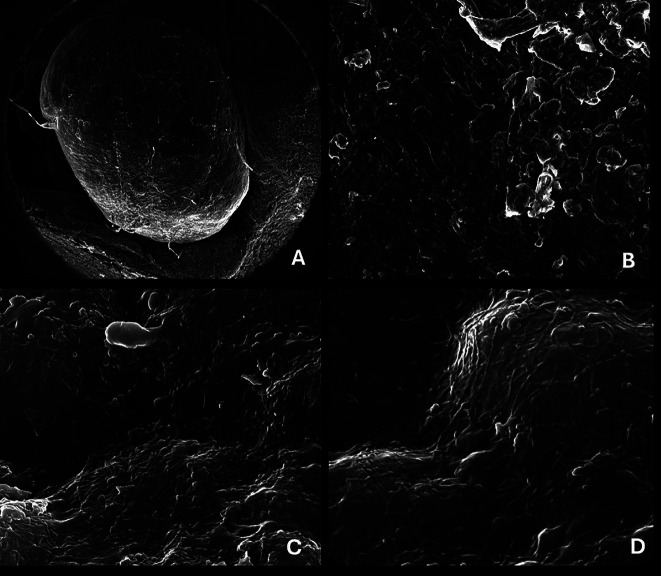
Electron microscopy of phleboliths. A) mag 61 x, B) mag 703 x, C) mag 30,000 x, D) mag 42 189 x.

The calcifications were studied with scan electron microscopy to know their chemical composition, observing mainly the presence of carbon, oxygen and calcium (Fig. 2). Regarding the tissue organization, it was possible to observe the development of filamentous aggregates of carbon macroparticles (nanofibers of amorphous carbon) (Fig. 3A-D), forming a compact structure of organic matter with inorganic aggregates (Fig. 3A).

## Discussion

The vascular anomalies are classified as vascular tumors and vascular malformations. Vascular tumors are more common in children, where congenital hemangiomas are the most frequent (3-5). Vascular malformations develop during adolescence or youth and venous malformations are the most common (2,5,6), which represent the second most common vascular anomaly, just after hemangioma (14).

Venous malformations are low-flux vascular anomalies; these lesions occur from childhood and are easily identified when they are located in superficial tissue. However, in deep locations they can only be identified with an image study in the zone with another purpose, as in the case presented here, which it was the diagnosis of an increased size of a malformation associated to puberty.

Venous malformations can present pain with time in addition to the volume increase during adolescence. Because they are low-flux malformations, small clots can develop secondary to trauma or venous stasis which, in turn, might favor the phlebolith formation (2,6,11,12). This is the case of the patient in this report, where nine phleboliths were formed with sizes ranging from 2 mm to 6 mm, which denotes the chronic phase of the lesion.

In venous malformations in children, it is difficult to observe phleboliths. However, the presence of phleboliths can help to determine, as a radiographic diagnostic, a vascular malformation, excluding the rest of vascular anomalies (9). Radiographically, the phleboliths are observed as round calcified masses (6) in 2% to 3% of the venous malformation cases (11). They can be single or multiple and the size is variable depending on the time of evolution of the lesion and the venous stasis. In the present case, nine circular hyperdense foci (calcified foci) were identified by CAT scan, with sizes ranging from 2 mm to 6 mm in diameter. The two years evolution that the patient referred to, and the size of the lesion in the moment of diagnosis, support the assumption of a direct relationship between the time of evolution and the size of the lesion with the development of phleboliths. As observed in the results from the electron microscopy, the phleboliths are mainly constituted of organic matter, organized in filaments that aggregate until they form a dense mass that will continue growing in a chronic way.

Phleboliths are described in literature as thrombotic calcifications (11). With electron microscopy, it was possible to identify the constitution of the filamentous aggregates in this clinical case. They were composed by carbon macroparticles (amorphous carbon nanofibers) with a highly organized development that causes compression of the organic matter. This structure allowed them to aggregate progressively more filaments and inorganic matter deposits, favoring the progressive growth of the phlebolith.

## Conclusions

The vascular anomalies constitute a broad group of lesions, where the most common is the congenital hemangioma, but the occurrence of venous malformations is also frequent at any age. As an important part of the evaluation both for the diagnosis and for the treatment of vascular anomalies, it is necessary to obtain a complete clinical history and a clinical evaluation of the lesion, and this should be complemented with imagenology studies, which allow the identification of its size and extent. The presence of calcifications must be considered a presumptuous diagnosis of venous malformation.

## Data Availability

The datasets used and analyzed in the current study are available from the corresponding author upon reasonable request.
